# Utilization of Ningxiang pig milk oligosaccharides by *Akkermansia muciniphila in vitro* fermentation: enhancing neonatal piglet survival

**DOI:** 10.3389/fmicb.2024.1430276

**Published:** 2024-06-12

**Authors:** Longlin Zhang, Zichen Wu, Meng Kang, Jing Wang, Bie Tan

**Affiliations:** ^1^Key Laboratory for Quality Regulation of Livestock and Poultry Products of Hunan Province, College of Animal Science and Technology, Hunan Agricultural University, Changsha, China; ^2^Yuelushan Laboratory, Changsha, China

**Keywords:** *Akkermansia muciniphila*, Ningxiang pig, milk oligosaccharides, piglet survival, short-chain fatty acids

## Abstract

*Akkermansia muciniphila* (*A. muciniphila*), an intestinal symbiont residing in the mucosal layer, shows promise as a probiotic. Our previous study found that the abundance of *A. muciniphila* was significantly higher in Ningxiang suckling piglets compared to other breeds, suggesting that early breast milk may play a crucial role. This study examines *A. muciniphila*’s ability to utilize Ningxiang pig milk oligosaccharides. We discovered that *A. muciniphila* can thrive on both Ningxiang pig colostrum and purified pig milk oligosaccharides. Genetic analysis has shown that *A. muciniphila* harbors essential glycan-degrading enzymes, enabling it to effectively break down a broad spectrum of oligosaccharides. Our findings demonstrate that *A. muciniphila* can degrade pig milk oligosaccharides structures such as 3′-FL, 3′-SL, LNT, and LNnT, producing short-chain fatty acids in the process. The hydrolysis of these host-derived glycan structures enhances *A. muciniphila*’s symbiotic interactions with other beneficial gut bacteria, contributing to a dynamic microbial ecological network. The capability of *A. muciniphila* to utilize pig milk oligosaccharides allows it to establish itself in the intestines of newborn piglets, effectively colonizing the mucosal layer early in life. This early colonization is key in supporting both mucosal and metabolic health, which is critical for enhancing piglet survival during lactation.

## 1 Introduction

Ensuring the survival and robust health of newborn piglets is crucial for the economic sustainability of pig farming (Heuß et al., 2019). Notably, approximately 15–20% of piglets succumb within the first 3 days post-birth, primarily due to inadequate immune development and environmental stressors (Bæk et al., 2019; Farmer and Edwards, 2022). Finding certain measures to address these challenges is critical for enhancing piglet survival and overall production efficiency.

Colostrum plays a vital dual role in the early life of piglets. The intake of colostrum within the first 24 h post-birth is essential, not only nourishing the piglets but also fortifying their systemic immune systems, thereby promoting their growth and increasing their resilience against infections (Ogawa et al., 2016; Kogut and Zhang, 2022). Overall, it serves as an indispensable source of nutrients and crucially shapes the neonatal gut microbiota through its rich content of milk oligosaccharides (MOs) (Zivkovic et al., 2011). MOs are composed of a linear or branched backbone containing galactose, *N*-acetylglucosamine, and glucose, which can be decorated with fucose or sialic acid residues (Li et al., 2022). Although these complex carbohydrates are indigestible by piglets themselves, they are vital for the proliferation of specific beneficial bacteria. This strategic nourishment results in a beneficial microbiota composition that bolsters health and disease resistance, a reflection of evolutionary adaptations aimed at optimizing both infant survival and maternal health.

The Ningxiang breed is known among China’s four famous pig breeds for its resilience and notably low rates of diarrhea in neonates (Wang et al., 2014; Ma et al., 2022). In our previous investigations, we observed that *Akkermansia muciniphila* (*A. muciniphila*) was notably more prevalent in Ningxiang suckling piglets compared to other breeds (Unpublished data). This Gram-negative anaerobe from the Verrucomicrobia phylum is renowned for its ability to degrade mucin and colonize the mucus layer of the gastrointestinal tract and convert this polymer into mostly acetate and propionate (Dao et al., 2015; Zhang et al., 2019). The presence of *A. muciniphila* is beneficial for host metabolism and immunity, particularly in mitigating risks associated with chronic conditions such as obesity and diabetes (Zheng et al., 2023). The early colonization by *A. muciniphila*, evident from as early as the first month of life, plays a significant role in the developmental stages of the gut microbiota, facilitated by its ability to utilize MOs (Collado et al., 2007; Derrien et al., 2008).

Based on the above, we hypothesize that the significant presence of *A. muciniphila* in Ningxiang piglets is linked to its ability to metabolize pig milk oligosaccharides (PMOs), which, in turn, would have a beneficial effect on newborn piglets. In this study, an *in vitro* fermentation model to explore the fermentative behavior of *A. muciniphila* on PMOs derived from Ningxiang colostrum, examining growth kinetics and acid production as indicators of PMOs utilization. Through genomic analysis, we have identified the key enzymes responsible for the breakdown of these oligosaccharides, further reinforcing the potential role of *A. muciniphila* in promoting a healthy gut microbiota from the earliest stages of life. By understanding how specific microbial strains such as *A. muciniphila* exploit the unique glycobiome of breast milk, we pave the way for potential interventions aimed at fostering optimal microbial colonization patterns in early life, setting the stage for a healthier adult life.

## 2 Materials and methods

### 2.1 Materials

*A. muciniphila* DSM 22959 was purchased from the German Collection of Microorganisms and Cell Cultures (Braunschweig, Germany). 2′-fucosyllactose [2′-FL] (purity≥95%), 3-fucosyllactose [3′-FL] (purity≥95%), 3′-siallylactose [3′-SL] (purity≥95%), 6′-sialyllactose [6′-SL] (purity≥95%), lacto-*N*-tetraose [LNT] (purity≥95%) and lacto-*N*-neotetraose [LNnT] (purity≥95%) were kindly provided by Zhuo Wang and Siming Jiao, from State Key Laboratory of Biochemical Engineering, Institute of Process Engineering, Chinese Academy of Sciences (Beijing, China). All the reagents used for this study were of analytical grade.

### 2.2 Bacterial growth curves

*A. muciniphila* DSM 22959 growth were performed with cells grown in brain heart infusion broth medium (BHI) (1 mL per replicate) broth at 37°C in anaerobic chamber (Whitley A35 anaerobic workstation). Cells were inoculated in BHI broth supplemented with Ningxiang-pig colostrum (10% v/v), Ningxiang-pig purified PMOs (1 mg/mL), neutral trioses (2′-FL and 3′-FL), tetraoses (LNT, LNnT), and acidic trioses (3′-SL, 6′-SL) (10 mM respectively). Medium without any supplementation was included as a control. Cells were incubated anaerobically at 37°C until reaching stationary phase (48 h).

### 2.3 PMOs extraction

The PMOs were isolated and purified as previously described, with minor modification (Barile et al., 2010). Briefly, frozen milk samples were completely thawed, and a 20 mL aliquot of each sample was mixed with an equal volume of nanopure water and centrifuged at 14,000 × *g* in a microfuge for 30 min at 4°C to remove lipids. The top fat layer was removed, and 4 volumes of chloroform: methanol (2:1, vol/vol) were added, vigorously mixed, and the resulting emulsion was centrifuged at 4,000 × *g* for 30 min at 4°C. The upper methanol layer containing PMOs was transferred to a tube, 2 volumes of cold ethanol were added, and the solution was frozen for 1 h at −30°C, followed by centrifugation for 30 min at 4,000 × *g* and 4°C to precipitate the denatured protein. The supernatant (PMOs-rich fraction) was collected and further purified. Oligosaccharides were purified from the mixture by GCC-SPE. Prior to use, each GCC-SPE cartridge was activated with 3 column volumes of 80% acetonitrile (ACN) and 0.1% trifluoroacetic acid (TFA, vol/vol) and equilibrated with 3 column volumes of nanopure water. The carbohydrate-rich solution was loaded onto the cartridge, and salts and mono- or disaccharides were removed by washing with 10 column volumes of nanopure water. Then, it was eluted with a solution of 40% ACN with 0.1% TFA (vol/vol) in water and the solution was frozen for 1 h at −80°C, with freeze-dried using a lyophilizer overnight to collected purified PMO.

### 2.4 Carbohydrate-active enzyme (CAZyme) annotation

CAZymes within *A. muciniphila* DSM 22959 (NCBI Reference Sequence: GCF_008000975.1) was annotated using the dbCAN2 metaserver.^1^ CAZymes identified by at least two out of three tools were considered for further analysis.

### 2.5 Quantitative real-time PCR (qPCR)

The abundance of *A. muciniphila* was determined by qPCR as described previously10. Cells (1 mL) were harvested at 21,000 × g for 15 min. DNA extractions were performed using the MasterPure™ Gram Positive DNA Purification Kit (Epicentre, Lucigen, United States). DNA concentrations were measured fuorometrically (Qubit dsDNA BR assay, Invitrogen) and adjusted to 1 ng/μL prior to use as the template in qPCR. Primers targeting the 16S rRNA gene of *A. muciniphila* (5′-CAGCACGTGAAGGTGGGGAC-3′ and 5′-CCTTGCGGTTGGCTTCAGAT-3′; 327 bp) were used for quantification. A standard curve was prepared with nine standard concentrations from 100 to 108 gene copies/μL. qPCR was performed in triplicate with iQ SYBR green supermix (Bio-Rad, United States) in a total volume of 10 μL prepared with primers at 500 nM in 384-wells plates with the wells sealed with optical sealing tape. Amplification was performed with an iCycler (Bio-Rad): one cycle of 95°C for 10 min; 40 cycles of 95°C for 15 s, 60°C for 20 s, and 72°C for 30 s each; one cycle of 95°C for 1 min; and a stepwise increase of temperature from 60 to 95°C (at 0.5°C per 5 s) to obtain melt curve data. Data were analyzed using Bio-Rad CFX Manager 3.0. The copy number was corrected for the DNA concentration and for the number of 16S rRNA genes encoded in *A. muciniphila*’s genome.

### 2.6 SCFAs analysis

The concentrations of SCFAs in *A. muciniphila* fermentation were analyzed using the gas chromatographic (GC) method. Briefly, 1 mL *A. muciniphila* fermentation was first centrifuged at 15,000 × *g* for 10 min at 4°C. The samples were acidified with 25% metaphosphoric acid at a ratio of 1:5 for 30 min on ice. Samples were injected into a GC 8890 series gas chromatograph (Agilent, United States) for detection.

### 2.7 Statistical analysis

The data were analyzed using SPSS 26.0 statistical software (ver. 26.0 for Windows, SPSS Inc., Chicago, IL, United States). All data were analyzed by Student’s *t*-test and expressed as means with their standard errors. And ^*^p < 0.05, ^**^p < 0.01, and ^***^p < 0.001, Colostrum group compared with the CON group. ^#^*p* < 0.05, ^##^*p* < 0.01, and ^###^*p* < 0.001 compared with the same treatment at different time.

## 3 Results

### 3.1 *A. muciniphila* DSM 22959 growth in Ningxiang-pig colostrum

**Figure 1:**
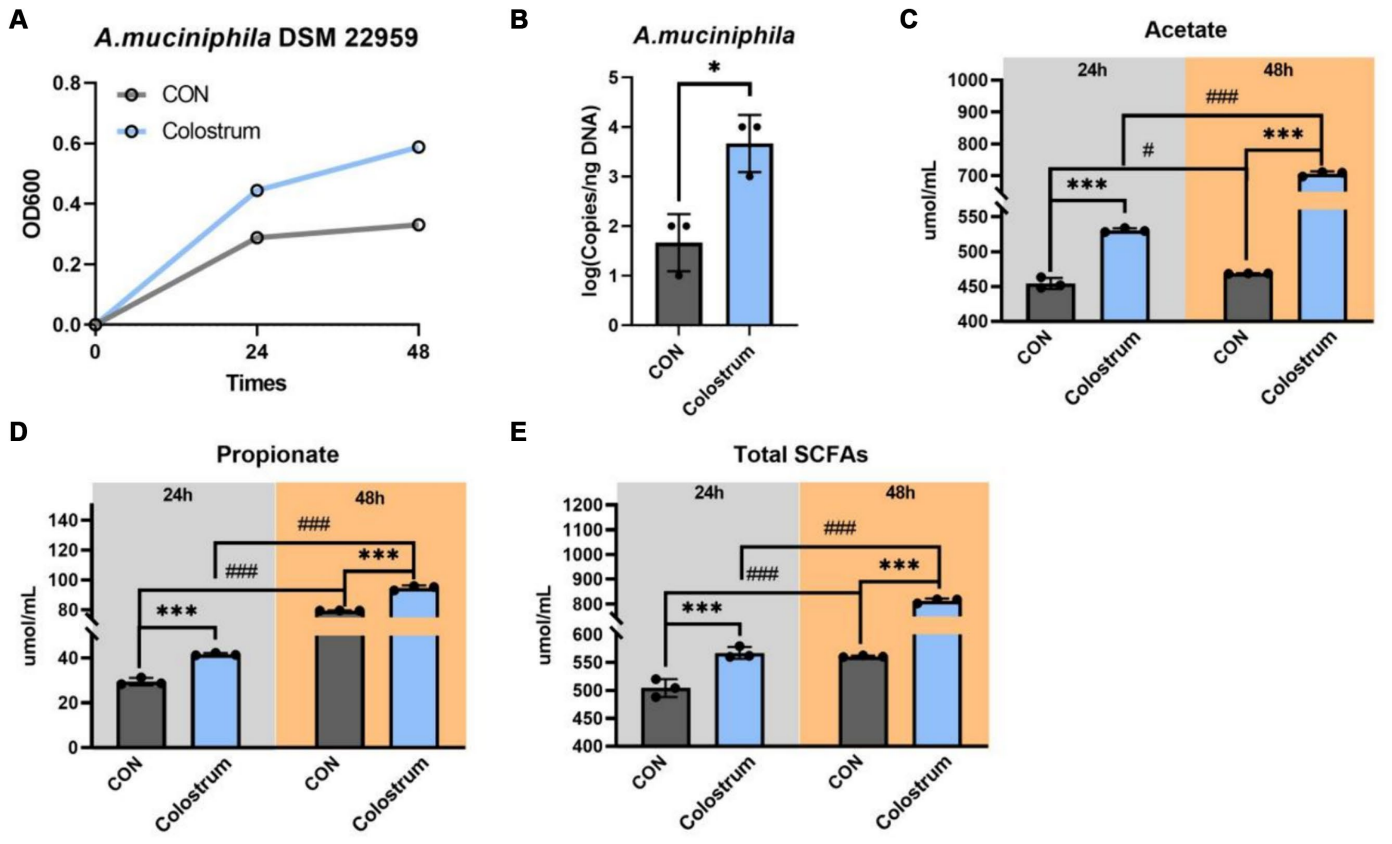
*Akkermansia muciniphila* DSM 22959 growth in Ningxiang-pig colostrum. **(A)** *A. muciniphila* DSM 22959 growth was assessed over a 48 h period by measuring optical density at a wavelength of 600 nm (OD600). Bacteria were grown in brain heart infusion broth medium (BHI), or BHI supplemented with Ningxiang-pig colostrum (10% v/v). **(B)** *A. muciniphila* DSM 22959 was grown *in vitro* in the absence or presence of 10% v/v Ningxiang-pig colostrum until 48 h of growth; *A. muciniphila* was quantified by qPCR. **(C–E)** Changes in SCFA levels in fermentation solutions. Data are expressed as the mean ± standard deviation (*n* = 3). ^*^p < 0.05, and ^***^p < 0.001, Colostrum group compared with the CON group. ^#^*p* < 0.05, and ^###^*p* < 0.001 compared with the same treatment at different time.

Incubation of *A. muciniphila* DSM 22959 on Ningxiang-pig colostrum resulted in growth (Figure 1A). The results showed that presence of Ningxiang-pig colostrum in the basal medium (10% v/v) resulted in a modest increase in *A. muciniphila* growth *in vitro* (Figure 1A). And basal medium supplemented with Ningxiang-pig colostrum (10% v/v) significantly increased the cell number of *A. muciniphila in vitro* at 48 h (Figure 1B). Next, we determined the production of SCFAs by *A. muciniphila* DSM 22959 during fermentation of Ningxiang-pig colostrum at 24 and 48 h. Our results showed that fermentation of Ningxiang-pig colostrum significantly increased production of acetate, propionate and total SCFAs, regardless of whether it was 24 or 48 h (Figures 1C–E). Interestingly, the ratio of acetate increase (48 h/24 h) was superior in fermented Ningxiang pig colostrum compared to the increasing trend in the CON group (Figure 1C).

### 3.2 *A. muciniphila* DSM 22959 growth in Ningxiang-pig purified PMOs

**Figure 2:**
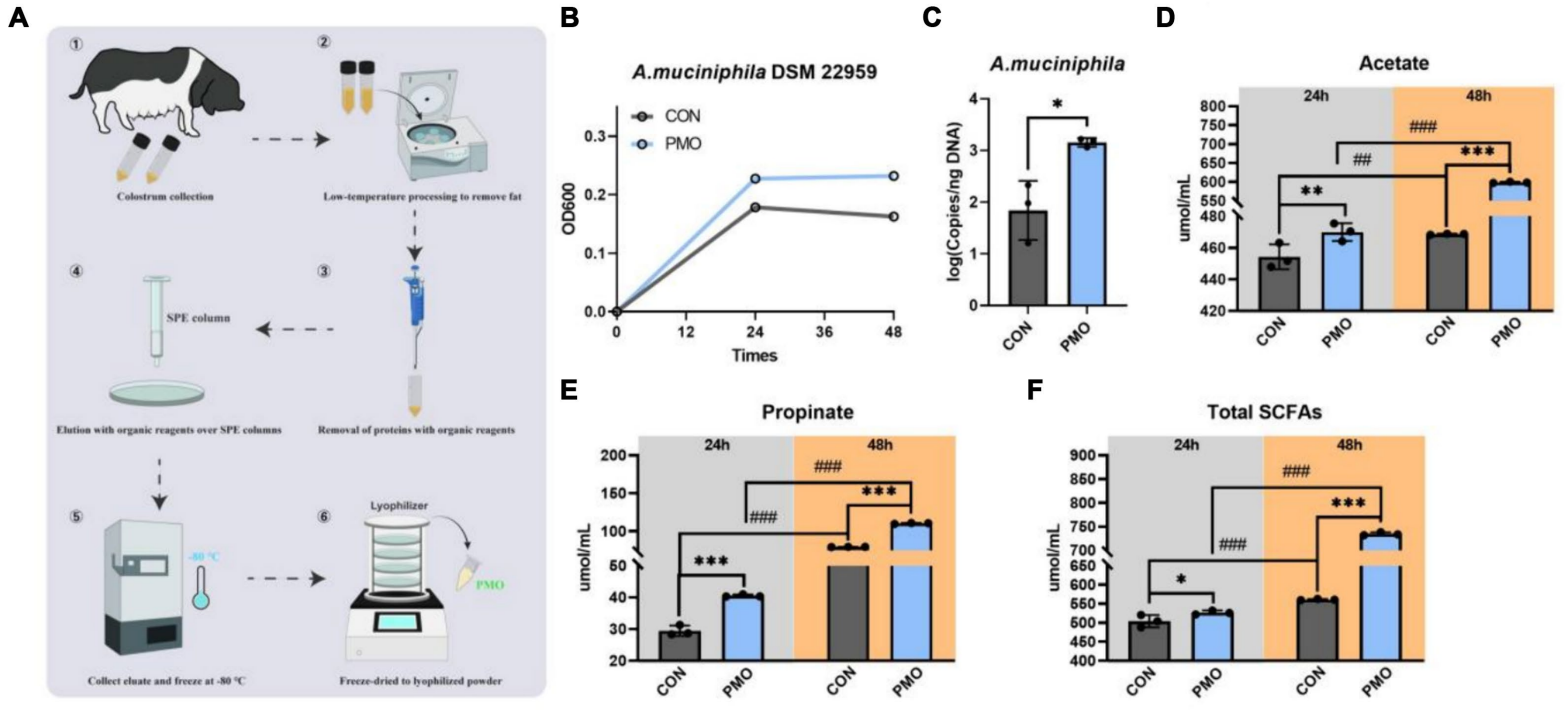
*Akkermansia muciniphila* DSM 22959 growth in Ningxiang-pig purified PMO. **(A)** Schematic illustration of Ningxiang-pig purified PMO preparation. **(B)** *A. muciniphila* DSM 22959 growth was assessed over a 48 h period by measuring optical density at a wavelength of 600 nm (OD600). Bacteria were grown in brain heart infusion broth medium (BHI), or BHI supplemented with Ningxiang-pig purified PMO (1 mg/mL). **(C)** *A. muciniphila* DSM 22959 was grown *in vitro* in the absence or presence of 1 mg/mL Ningxiang-pig purified PMO until 48 h of growth; *A. muciniphila* was quantified by qPCR. **(D–F)** Changes in SCFA levels in fermentation solutions. Data are expressed as the mean ± standard deviation (*n* = 3). ^*^p < 0.05, ^**^p < 0.01, and ^***^p < 0.001, Colostrum group compared with the CON group. ^##^*p* < 0.01, and ^###^*p* < 0.001 compared with the same treatment at different time.

Human milk oligosaccharides (HMOs) play an important role in the early nutrition of nursing infants and can act as substrates to support bacterial growth and thus dominate the gut early in life. In order to determine whether *A. muciniphila* DSM 22959 can utilize breast milk oligosaccharides to promote its own growth, we purified PMOs from the Ningxiang-pig colostrum (Figure 2A). Our results showed that supplemented with PMOs (1 mg/mL) increased the proliferation rate and significantly increased the cell number of *A. muciniphila in vitro* at 48 h (Figures 2B,C). Additionally, PMOs also significantly increased production of acetate, propionate and total SCFAs, regardless of whether it was 24 or 48 h (Figures 2D–F).

### 3.3 Identification of CAZymes in *A. muciniphila* DSM 22959 genome

**Figure 3:**
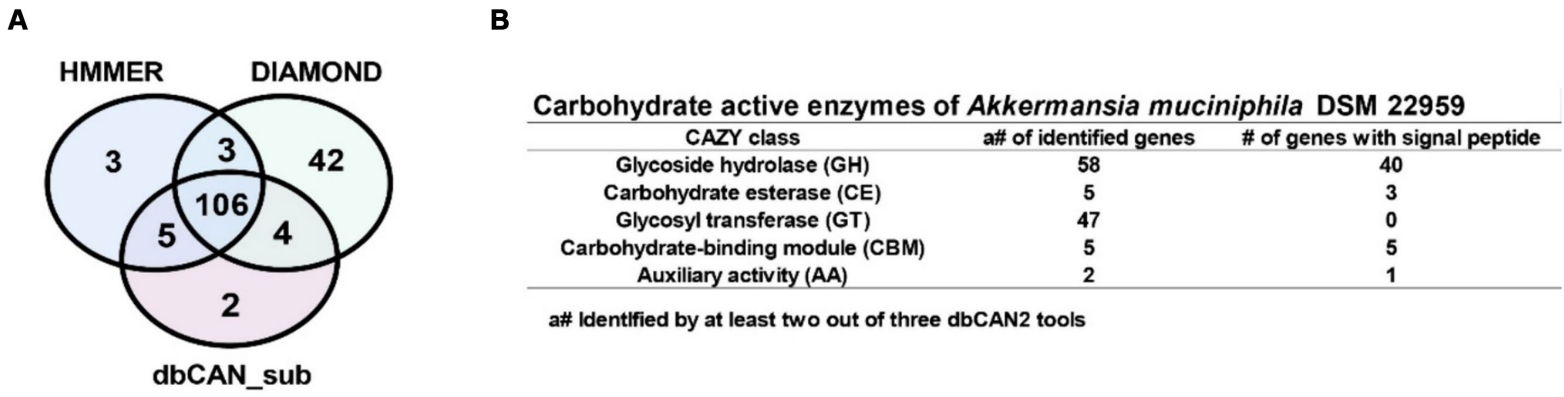
Identification of carbohydrate active enzymes (CAZymes) in *A. muciniphila* DSM 22959 genome. **(A)** Proportional Venn diagram showing the number of identified CAZyme encoding genes using the Diamond, HMMER and dbCAN_sub tools that are part of the dbCAN2 metaserver analysis. **(B)** The table shows the CAZymes and the presence of signal peptides, separated by CAZyme class, that were identified by at least two out of three dbCAN2 tools.

To investigate how *A. muciniphila* DSM 22959 can utilize complex PMO, we interrogated the available *A. muciniphila* DSM 22959 genome for the presence of CAZymes using the dbCAN2 metaserver pipeline for automated CAZyme annotation. This pipeline uses a combination of three different annotation tools (Diamond, HMMER and dbCAN_sub) to increase accuracy in predicting and annotating CAZymes within bacterial genomes. This analysis revealed a total of 165 putative ORFs with putative CAZy domains (Figure 3A).

For increased accuracy, we discarded hits that were not identified by at least two out of three tools for further analysis. Among the remaining 118 ORFs multiple types of CAZy domains could be detected, such as glycoside hydrolase (GH), carbohydrate esterase (CE), glycosyl transferase (GT) domains, and carbohydrate-binding modules (CBM), with some genes encoding a combination of multiple domains (Figure 3B). Additionally, most of the putative ORFs belong to the GH and GT classes, and also encode signal peptides, suggests that *A. muciniphila* DSM 22959 was able to utilize multiple MOs.

### 3.4 The genome of *A. muciniphila* predicts the presence of a wide range of MOs targeting CAZymes

**Figure 4:**
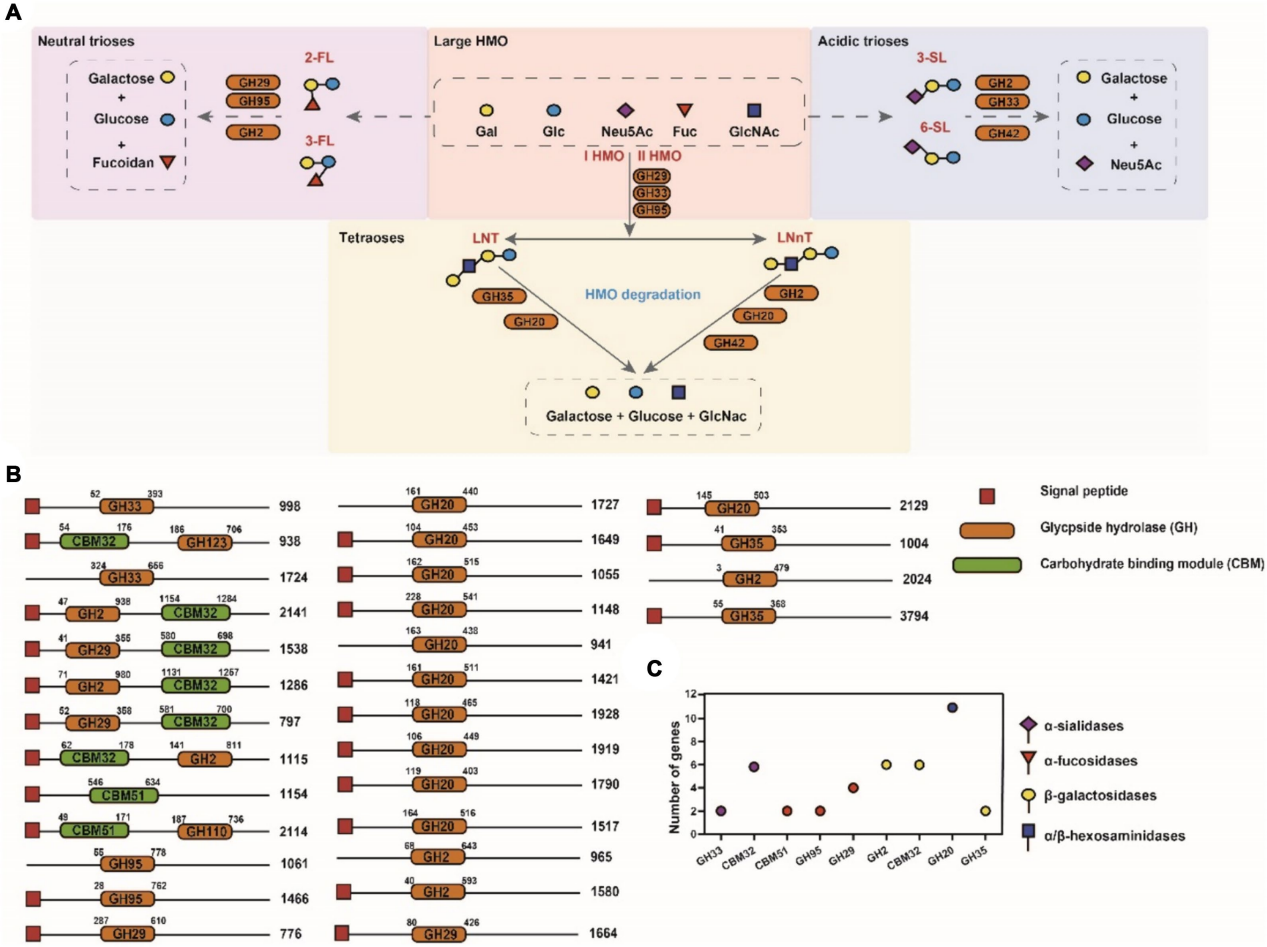
Domain architecture of putative MO targeting CAZymes in genome of *A. muciniphila* DSM 22959. **(A)** Simplified schematics of pathways involved in main HMO degradation (acidic trioses, neutral trioses and tetraoses). **(B)** CAZymes are divided in groups based upon their predicted enzyme activity: from top to bottom, *α*-sialidases, *α*-fucosidases, *β*-galactosidases and *α*/*β*-hexosaminidases. The displayed domains are those identified by HMMER, in number of amino acids, indicated at the right side of each protein. **(C)** The number of putative MO targeting CAZymes of *A. muciniphila* DSM 22959.

We performed further analysis on the *A. muciniphila* CAZy domains to identify the active enzymes that could contribute to the degradation of PMO (Figure 4A). Closer inspection of the Research Topic of CAZymes identified in the *A. muciniphila* DSM 22959 genome revealed at least 30 glycoside hydrolases that were predicted to utilize MOs, of which the majority contains a signal peptide (Figures 4B,C). Among the 30 glycoside hydrolases are two sialidases (GH33) and four fucosidases (GH29/95), which target the monosaccharides often found at terminal positions of MOs, and six different galactosidases (GH2) and 10 hexosaminidases (GH20/GH40), which are predicted to hydrolyze the underlying glycosidic linkages. The gene structure of the 30 putative enzymes, with the locations of the predicted glycoside hydrolase domains and additional domains clearly demonstrates the great diversity in domains, domain organization and predicted protein sizes – also between genes predicted to encode the same class of enzymes (Figure 4B). Transcription and translation of the entire repertoire of MO-targeting CAZymes would allow degradation of the wide variety of linkages commonly found within MOs. These data demonstrate that *A. muciniphila* DSM 22959 has the enzymatic capacity to utilize a broad range of MOs as well as their constituents.

### 3.5 *A. muciniphila* DSM 22959 growth in acidic trioses, neutral trioses and tetraoses

**Figure 5:**
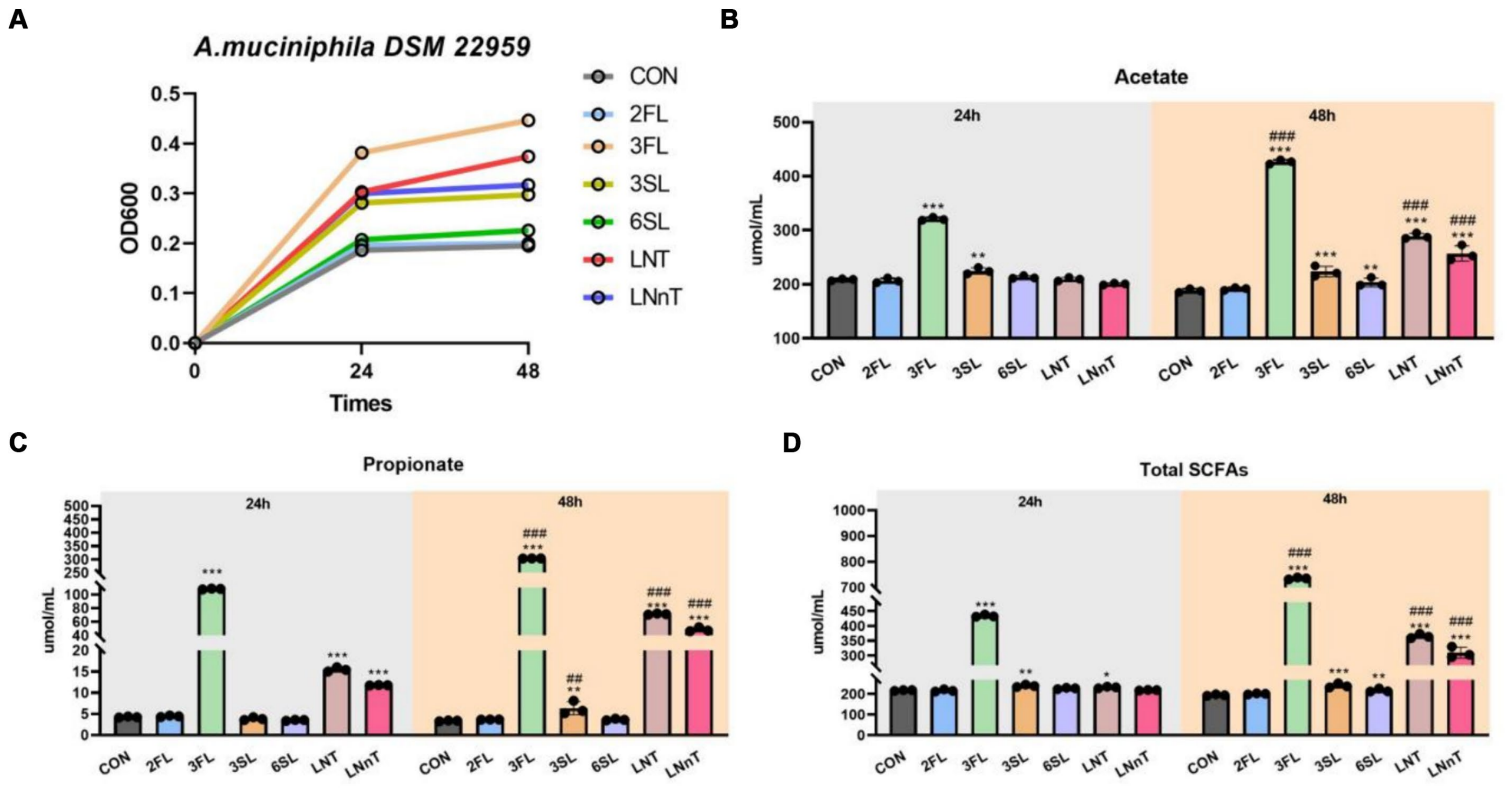
*A. muciniphila* DSM 22959 growth in acidic trioses, neutral trioses and tetraoses. **(A)** *A. muciniphila* DSM 22959 growth was assessed over a 48 h period by measuring optical density at a wavelength of 600 nm (OD600). Bacteria were grown in brain heart infusion broth medium (BHI), or BHI supplemented with neutral trioses (2-fucosyllactose [2-FL] and 3-fucosyllactose [3-FL]), tetraoses (lacto-*N*-tetraose [LNT], lacto-*N*-neotetraose [LNnT]), and acidic trioses (3-siallylactose [3-SL], 6-sialyllactose [6-SL]) (10 mM respectively). **(B–D)** Changes in SCFA levels in fermentation solutions. Data are expressed as the mean ± standard deviation (*n* = 3). ^*^p < 0.05, ^**^p < 0.01, and ^***^p < 0.001, Colostrum group compared with the CON group. ^##^*p* < 0.01, and ^###^*p* < 0.001 compared with the same treatment at different time.

We next sought to investigate which PMOs structures were utilized by *A. muciniphila* during the growth on colostrum. The results showed that *A. muciniphila* DSM 22959 was able to utilize acidic trioses, neutral trioses and tetraoses to grow, especially 3′-FL (Figure 5A). Interestingly, the two type of tetraoses (LNT, LNnT) can all support the growth of *A. muciniphila* DSM 22959, whereas only one type of acidic trioses (3′-FL) and neutral trioses (3′-SL) showed better utilization. Additionally, supplementation with 3′-FL significantly increased production of acetate, propionate and total SCFAs, regardless of whether it was 24 or 48 h (Figures 5B–D).

## 4 Discussion

The survival rate of newborn piglets significantly impacts the economic viability of the pig industry, with a major focus in animal science being how to enhance piglet vigor through nutrition (Ji et al., 2019; Xu et al., 2019). Based on previous research findings, *A. muciniphila* was able to act as an early characterizing gut microbiota in Ningxiang piglets. *A. muciniphila* consumes mucin and exhibits beneficial effects on the host. Considering the structural similarities between MOs and intestinal mucus, it is plausible that the presence of PMOs in breast milk significantly contributes to the early colonization of *A. muciniphila* in piglets. Studies have identified negative associations between *A. muciniphila* and various diseases such as inflammatory bowel disease, diabetes, and hypertension, and its role in modulating responses to PD-1 blockers in cancer therapy (Derosa et al., 2022). Innovations in bacterial preparation, such as the use of cryopasteurized *A. muciniphila*, have shown metabolic improvements in mice, attributed to the activity of Amuc-1100 protein. This protein interacts with Toll-like receptor 2 to enhance intestinal barrier function and exerts probiotic benefits (Depommier et al., 2019). Furthermore, oral administration of *A. muciniphila* has been demonstrated to boost intestinal stem cell proliferation and promote the differentiation of Paneth and goblet cells, facilitating the regeneration of intestinal epithelia (Kim et al., 2021). Meanwhile, it has been showed that *A. muciniphila* can protect the intestinal health of weaned piglets from damage caused by ETEC infection (Lan et al., 2024).

Colostrum is indispensable for piglet development, serving not only as a vital nutrient source but also significantly shaping the neonatal gut microbiota through its rich content of MOs. Although piglets cannot digest these complex carbohydrates, they are crucial for the proliferation of beneficial bacteria. MOs, primarily undergraded by gastric acid and digestive enzymes, reach the hindgut to undergo microbial fermentation. As a principal carbohydrate source selectively utilized by the gut microbiota, MOs profoundly influence the microbial composition of the host’s gut microbiota and are deemed crucial regulators in the development of the neonatal gut microbiota. Extensive studies have shown that MOs provide substrates for the gut microbiota, thereby facilitating the colonization and survival of specific gut microbiota. The complete degradation of MOs, featuring diverse molecular structures, necessitates a variety of glycoside hydrolases or membrane transporters present in the neonatal gut microbiota, essential for the uptake, metabolism, and utilization of MOs by gut microbiota. Recent research has highlighted that certain *Bifidobacteria* spp. can utilize specific MOs in breast milk for their colonization and growth, thus benefiting the host’s immune development, particularly in early life (Lawson et al., 2020; Heiss et al., 2021; Henrick et al., 2021). Furthermore, the mechanisms of MOs degradation and utilization by *Bifidobacteria* spp. are categorized into strategies dependent on bacterial intracellular membrane translocation and those reliant on extracellular glycosidases (Katayama, 2016). Other bacteria like *Bacteroides fragilis* and *Bacteroides vulgatus* also exhibit high MOs metabolizing abilities (Yu et al., 2013), with *Bacteroides fragilis* possessing genes crucial for the metabolism of mucus-sugar conjugates (Marcobal et al., 2011). Our study’s detailed genomic analysis has identified specialized CAZymes in *A. muciniphila*, which are specifically adapted for breaking down PMOs from Ningxiang colostrum. These enzymes not only facilitate the ability of gut microbiota to establish and maintain a healthy microbial balance within the neonatal piglet gut but also enable the effective fermentation of a broad range of PMOs. This process highlights *A. muciniphila*’s critical role in shaping the gut microbiota of neonatal piglets, underscoring its evolutionary adaptability and ecological significance, similar to its beneficial functions in human infants.

The MOs have an enormous molecular weight that cannot be directly utilized by cell, but can be catabolized by gut microbiota, then producing SCFAs to respond to various life activities, such as immune regulation and glucose homeostasis (Zhu et al., 2020; Zhang et al., 2021; Chen et al., 2023). SCFAs are the main products of prebiotic fermentation, and the production of these metabolites depends on both dietary fiber types and gut microbiota composition (Bridgman et al., 2017). There is increasing evidence that gut microbial metabolites have wide systemic effects on host through acting as signaling molecules (Gasaly et al., 2021). SCFAs have been implicated in immune system development by modulating the production of immune mediators and regulating the activation and differentiation of immune cells (Yao et al., 2022; Liu et al., 2023). To investigate the metabolic performance of colostrum, purified PMOs and specific MOs fermentation by *A. muciniphila* dominant inocula, the yield of SCFAs were quantified. Our results showed that fermentation of Ningxiang-pig colostrum and purified PMOs significantly increased production of acetate, propionate and total SCFAs, regardless of whether it was 24 or 48 h. The production of SCFAs by *A. muciniphila* during the fermentation of pig colostrum oligosaccharides not only supports its own growth but also mediates significant health benefits for the host. In piglets, these SCFAs can enhance gut integrity, reduce pathogen colonization, and improve nutrient absorption—factors that are crucial for early development and long-term health (Li et al., 2021; Qin et al., 2023). By extending these findings, we can hypothesize that strategic dietary interventions in livestock could replicate these benefits, potentially transforming approaches to neonatal care in pig farming.

In conclusion, this study demonstrated the ability of *A. muciniphila* to grow on Ningxiang PMOs thanks to its expression of a set of PMOs-degrading enzymes using an *in vitro* fermentation model. The fermentation of Ningxiang pig colostrum by *A. muciniphila* degraded acidic trioses, neutral trioses and tetraoses (the mainly PMOs). Our study offers the possibility of controlling colostrum composition or adding prebiotics to piglet diets to promote beneficial bacteria, such as *A. muciniphila*. Such strategies could lead to enhanced survival rates, improved immune functions, and greater resistance to gastrointestinal diseases in neonatal livestock, underscoring the potential of targeted microbial interventions in agricultural settings.

## Data availability statement

The original contributions presented in the study are included in the article/supplementary material, further inquiries can be directed to the corresponding authors.

## Author contributions

LLZ: Formal analysis, Visualization, Writing – original draft, Writing – review & editing. ZCW: Methodology, Software, Writing – review & editing. MK: Supervision, Validation, Writing – review & editing. JW: Conceptualization, Funding acquisition, Writing – review & editing. BET: Conceptualization, Funding acquisition, Writing – review & editing.

## Footnotes

^1^ http://bcb.unl.edu/dbCAN2/
